# Supplementary material for: Mitochondrial sequence data reveal population structure within Pustulosa pustulosa
Source: PeerJ. 2023 Oct 2;11:e15974. doi: 10.7717/peerj.15974 (PMC10552767; doi:10.7717/peerj.15974)
Supplement: Supplemental Information 1 [file peerj-11-15974-s001.pdf]

Electronic Supplemental information

Mitochondrial sequence data reveal population structure within *Pustulosa pustulosa*

David Rodriguez[1*], Stephen Forrest Harding[1], Shashwat Sirsi[1], Kelly McNichols-O'Rourke[2], Todd Morris[2], Michael R.J. Forstner[1], and Astrid N. Schwalb[1]

[1]Department of Biology, Texas State University; 601 University Drive, San Marcos, Texas 78666

[2]Fisheries and Oceans Canada, Great Lakes Laboratory for Fisheries and Aquatic Sciences, Burlington, ON L7S 1A1, Canada

*Corresponding author:

Email: drdz@txstate.edu

Table S1. Pairwise Φ_{ST} between river drainages

Table S2. Summary measures from tests for demographic expansion

Table S3. Pairwise Φ_{ST} between designated groups

Appendix S1. Samples analyzed at the ND1 locus

Table S1. Pairwise Φ_{ST} (lower diagonal) calculated using the distance method in Arlequin and between *Pustulosa* (*Cyclonaias*) *pustulosa sensu lato* grouped by river drainage (ND1 locus) and regional group designations. Negative values were treated as zero (italics) and font indicates low (grey), moderate (black), and high differentiation values (bold black). *P* values and standard deviations are given on the upper diagonal. Group designations and population numbers correspond to Fig. 1.

	I						II	III				IV		V		
River Drainages	Upper Mississippi (1)	Grand/E. Sydenham/Thames (2)	Ohio (3)	Arkansas/Missouri (4)	Lower Mississippi (5)	Red (6)	Pascagoula/Pearl (7)	Sabine (8)	Neches (9)	Trinity (10)	San Jacinto (11)	Brazos (12)	Colorado (13)	Guadalupe (14)	San Antonio (15)	Nueces (16)
1	*	0.03604+-0.0201	0.02703+-0.0139	0.22523+-0.0365	0.03604+-0.0201	0.00000+-0.0000	0.00000+-0.0000	0.00000+-0.0000	0.00000+-0.0000	0.00000+-0.0000	0.00000+-0.0000	0.00000+-0.0000	0.00000+-0.0000	0.00000+-0.0000	0.00000+-0.0000	0.00000+-0.0000
2	0.0141	*	0.00901+-0.0091	0.36036+-0.0542	0.06306+-0.0305	0.01802+-0.0121	0.00000+-0.0000	0.00000+-0.0000	0.00000+-0.0000	0.00000+-0.0000	0.00000+-0.0000	0.00000+-0.0000	0.00000+-0.0000	0.00000+-0.0000	0.00000+-0.0000	0.00000+-0.0000
3	0.0789	0.0791	*	0.14414+-0.0337	0.00000+-0.0000	0.00901+-0.0091	0.00901+-0.0091	0.00000+-0.0000	0.00000+-0.0000	0.00000+-0.0000	0.00000+-0.0000	0.00000+-0.0000	0.00000+-0.0000	0.00000+-0.0000	0.00000+-0.0000	0.00000+-0.0000
4	0.0119	0.0042	0.0317	*	0.06306+-0.0273	0.18919+-0.0212	0.16216+-0.0353	0.00000+-0.0000	0.00000+-0.0000	0.00000+-0.0000	0.00000+-0.0000	0.00000+-0.0000	0.00000+-0.0000	0.00000+-0.0000	0.00901+-0.0091	0.04505+-0.0203
5	0.0207	0.0126	0.1060	0.0325	*	0.45045+-0.0489	0.00000+-0.0000	0.00000+-0.0000	0.00000+-0.0000	0.00000+-0.0000	0.00000+-0.0000	0.00000+-0.0000	0.00000+-0.0000	0.00000+-0.0000	0.00000+-0.0000	0.00000+-0.0000
6	0.0256	0.0206	0.0652	0.0305	*-0.0001*	*	0.00000+-0.0000	0.00000+-0.0000	0.00000+-0.0000	0.00000+-0.0000	0.00000+-0.0000	0.00000+-0.0000	0.00000+-0.0000	0.00000+-0.0000	0.00000+-0.0000	0.00000+-0.0000
7	0.2358	0.2418	0.1948	0.0742	0.3019	0.2873	*	0.00000+-0.0000	0.00000+-0.0000	0.00000+-0.0000	0.00000+-0.0000	0.00000+-0.0000	0.00000+-0.0000	0.00000+-0.0000	0.00000+-0.0000	0.02703+-0.0139
8	**0.6105**	**0.6190**	**0.6196**	**0.5496**	**0.6569**	**0.6685**	**0.5727**	*	0.07207+-0.0264	0.03604+-0.0148	0.11712+-0.0333	0.00000+-0.0000	0.00000+-0.0000	0.00000+-0.0000	0.00000+-0.0000	0.00000+-0.0000
9	**0.6173**	**0.6307**	**0.6480**	**0.6040**	**0.6572**	**0.6575**	**0.5848**	0.054	*	0.00901+-0.0091	0.02703+-0.0194	0.00000+-0.0000	0.00000+-0.0000	0.00000+-0.0000	0.00000+-0.0000	0.00000+-0.0000
10	**0.4627**	**0.4598**	**0.4131**	0.3469	**0.4861**	**0.4830**	0.3520	0.0763	0.0699	*	0.85586+-0.0281	0.00000+-0.0000	0.00000+-0.0000	0.00000+-0.0000	0.00000+-0.0000	0.00000+-0.0000
11	**0.4611**	**0.4606**	0.3876	0.3151	**0.4892**	**0.4871**	0.3218	0.0762	0.0618	*-0.0475*	*	0.00000+-0.0000	0.00000+-0.0000	0.00000+-0.0000	0.00000+-0.0000	0.00000+-0.0000
12	0.3371	0.3350	**0.4697**	**0.4496**	**0.4161**	**0.4167**	**0.5861**	**0.8454**	**0.7561**	**0.6203**	**0.6758**	*	0.10811+-0.0297	0.00000+-0.0000	0.00000+-0.0000	0.00000+-0.0000
13	0.3197	0.3148	0.3722	0.3755	0.3724	0.3563	**0.4954**	**0.7523**	**0.7233**	**0.5979**	**0.6292**	0.0218	*	0.00000+-0.0000	0.00000+-0.0000	0.00000+-0.0000
14	**0.4962**	**0.4923**	**0.4831**	**0.4110**	**0.5285**	**0.5213**	0.3520	**0.5660**	**0.5913**	**0.4706**	**0.4675**	**0.6179**	**0.6171**	*	0.22523+-0.0339	0.00000+-0.0000
15	**0.4362**	**0.4311**	**0.4155**	0.3205	**0.4763**	**0.4759**	0.2875	**0.5549**	**0.6024**	**0.4250**	**0.4166**	**0.6279**	**0.6131**	0.0065	*	0.05405+-0.0201
16	0.2647	0.2494	0.2226	0.1400	0.2753	0.2631	0.1729	**0.4592**	**0.5313**	0.3528	0.3328	**0.4247**	**0.4505**	0.1489	0.0776	*

Table S2. Summary measures from tests for spatial expansion in Arlequin using ND1 sequence data. Group II was excluded from these analyses owing to low sample sizes.

Group	Sum of Squared deviation	P(Sim. Ssd >= Obs. Ssd)	Harpending's Raggedness index	P(Sim. Rag. >= Obs. Rag.)	TD	P	Fu's	P
I	0.002	0.290	0.012	0.460	-2.230	< 0.001	-25.213	< 0.001
III	0.008	0.010	0.022	0.290	-2.080	0.001	-25.834	< 0.001
IV	0.006	0.850	0.045	0.910	-2.262	0.002	-13.332	0.001
V	0.015	0.030	0.027	0.510	-1.431	0.042	-24.920	< 0.001

Table S3. Pairwise Φ_{ST} calculated using the distance method in Arlequin and between designated groups of *Pustulosa* (*Cyclonaias*) *pustulosa using* ND1 sequence data. All comparisons are significant (P < 0.0001+- Std. Dev.). Group II was excluded from these analyses owing to low sample size.

Group	I	III	IV	V
I	-	<0.0001+-0.0	<0.0001+-0.0	<0.0001+-0.0
III	0.5496	-	<0.0001+-0.0	<0.0001+-0.0
IV	0.2967	0.6826	-	<0.0001+-0.0
V	0.3867	0.4892	0.5214	-

Appendix S1. Mussel samples showing phylogenetic affinity to *Pustulosa* (*Cyclonaias*) *pustulosa* at the mitochondrial ND1 locus, NA = data not available.

GenBank	ID	River	Basin	State/Province	Latitude	Longitude	Group	Dataset
OQ656460	AT026	Village Creek	Neches	Texas	30.261567	-94.159117	III	This study
OQ656461	AT028	Village Creek	Neches	Texas	30.261567	-94.159117	III	This study
OQ656462	AT036	Village Creek	Neches	Texas	30.249038	-94.126587	III	This study
OQ656463	AT039	Village Creek	Neches	Texas	30.249038	-94.126587	III	This study
OQ656464	AT042	Village Creek	Neches	Texas	30.243833	-94.124	III	This study
OQ656465	AT044	Village Creek	Neches	Texas	30.243833	-94.124	III	This study
OQ656466	AT046	Village Creek	Neches	Texas	30.243833	-94.124	III	This study
OQ656467	AT048	Village Creek	Neches	Texas	30.243833	-94.124	III	This study
OQ656468	AT049	Village Creek	Neches	Texas	30.243833	-94.124	III	This study
OQ656469	AT052	Village Creek	Neches	Texas	30.243833	-94.124	III	This study
OQ656470	AT053	Village Creek	Neches	Texas	30.243833	-94.124	III	This study
OQ656471	AT054	Village Creek	Neches	Texas	30.243833	-94.124	III	This study
OQ656472	AT078	Village Slough	Neches	Texas	30.239183	-94.11835	III	This study
OQ656473	AT093	Village Slough	Neches	Texas	30.239183	-94.11835	III	This study
OQ656474	AT098	Neches	Neches	Texas	30.25545	-94.104167	III	This study
OQ656475	AT205	Neches	Neches	Texas	30.26435	-94.110067	III	This study
OQ656476	AT206	Neches	Neches	Texas	30.26435	-94.110067	III	This study
OQ656477	AT207	Neches	Neches	Texas	30.26435	-94.110067	III	This study
OQ656478	AT216	Neches	Neches	Texas	30.211317	-94.101867	III	This study
OQ656479	AT217	Neches	Neches	Texas	30.211317	-94.101867	III	This study
OQ656480	AT218	Neches	Neches	Texas	30.211317	-94.101867	III	This study
OQ656481	AT219	Neches	Neches	Texas	30.211317	-94.101867	III	This study
OQ656482	AT224	Neches	Neches	Texas	30.173733	-94.1176	III	This study
AY158745	UAUC1083	Lake Corpus Christi	Nueces	Texas	NA	NA	V	Serb et al. 2003
AY158752	pust2587	Ouachita	Lower Mississippi	Arkansas	NA	NA	I	Serb et al. 2003
AY158753	pust2591	Ouachita	Lower Mississippi	Arkansas	NA	NA	I	Serb et al. 2003
AY158754	UAUC2590	Mississippi	Upper Mississippi	Missouri	NA	NA	I	Serb et al. 2003
AY158759	UAUC866	St. Croix	Upper Mississippi	Wisconsin	NA	NA	I	Serb et al. 2003
AY158762	pust658	Wolf	Lower Mississippi	Tennessee	NA	NA	I	Serb et al. 2003
AY158763	pust2441	Amite	Lower Mississippi	Louisiana	NA	NA	I	Serb et al. 2003
AY158764	mort1077	Big Cypress Bayou	Red	Texas	NA	NA	I	Serb et al. 2003
AY158766	UAUC2372	Amite	Lower Mississippi	Louisiana	NA	NA	I	Serb et al. 2003
AY158767	pust1019	Mississippi	Upper Mississippi	Illinois	NA	NA	I	Serb et al. 2003
AY158788	QREF405F	Spring	Arkansas	Kansas	NA	NA	I	Serb et al. 2003
OQ656483	BAH001	San Antonio River	Guadalupe	Texas	28.73764	-97.644465	V	This study
OQ656484	BAH004	San Antonio River	Guadalupe	Texas	28.73764	-97.644465	V	This study
OQ656485	BAH006	San Antonio River	Guadalupe	Texas	28.73764	-97.644465	V	This study
OQ656486	BAH012	San Antonio River	Guadalupe	Texas	28.73764	-97.644465	V	This study
OQ656487	BAH012a	San Antonio River	Guadalupe	Texas	28.73764	-97.644465	V	This study
OQ656488	BAH013	San Antonio River	Guadalupe	Texas	28.73764	-97.644465	V	This study
OQ656489	BAH014	San Antonio River	Guadalupe	Texas	28.73764	-97.644465	V	This study
OQ656490	BAH014a	San Antonio River	Guadalupe	Texas	28.73764	-97.644465	V	This study
OQ656491	BAH015	San Antonio River	Guadalupe	Texas	28.73764	-97.644465	V	This study
OQ656492	BAH015a	San Antonio River	Guadalupe	Texas	28.73764	-97.644465	V	This study
OQ656493	BAH016	San Antonio River	Guadalupe	Texas	28.73764	-97.644465	V	This study
OQ656494	BAH042	San Antonio River	Guadalupe	Texas	28.73764	-97.644465	V	This study
OQ656495	BAH043	San Antonio River	Guadalupe	Texas	28.73764	-97.644465	V	This study
OQ656496	BAH044	San Antonio River	Guadalupe	Texas	28.73764	-97.644465	V	This study
OQ656497	BAH045	Guadalupe River	Guadalupe	Texas	28.822267	-97.016892	V	This study
OQ656498	BAH046	San Antonio River	Guadalupe	Texas	28.73764	-97.644465	V	This study
OQ656499	BAH062	Guadalupe River	Guadalupe	Texas	29.150003	-97.316188	V	This study
OQ656500	BAH074	Guadalupe River	Guadalupe	Texas	29.150003	-97.316188	V	This study
OQ656501	BAH086	Guadalupe River	Guadalupe	Texas	28.822267	-97.016892	V	This study
OQ656502	BAH087	Guadalupe River	Guadalupe	Texas	28.822267	-97.016892	V	This study
OQ656503	BAH088	Guadalupe River	Guadalupe	Texas	28.822267	-97.016892	V	This study
OQ656504	BAH089	Guadalupe River	Guadalupe	Texas	28.822267	-97.016892	V	This study
OQ656505	BAH090	Guadalupe River	Guadalupe	Texas	28.822267	-97.016892	V	This study
OQ656506	BAH091	Guadalupe River	Guadalupe	Texas	28.822267	-97.016892	V	This study
OQ656507	BAH092	Guadalupe River	Guadalupe	Texas	28.822267	-97.016892	V	This study
OQ656508	BAH093	Guadalupe River	Guadalupe	Texas	28.822267	-97.016892	V	This study
OQ656509	BAH095	Guadalupe River	Guadalupe	Texas	28.822267	-97.016892	V	This study
OQ656510	BAH118	Guadalupe River	Guadalupe	Texas	28.822267	-97.016892	V	This study
OQ656511	BAH119	Guadalupe River	Guadalupe	Texas	28.822267	-97.016892	V	This study
OQ656512	BAH120	Guadalupe River	Guadalupe	Texas	28.822267	-97.016892	V	This study
OQ656513	BAH124	Guadalupe River	Guadalupe	Texas	28.822267	-97.016892	V	This study
OQ656514	BAH125	Guadalupe River	Guadalupe	Texas	28.822267	-97.016892	V	This study
OQ656515	BAH126	Guadalupe River	Guadalupe	Texas	28.822267	-97.016892	V	This study
OQ656516	BAH131	Guadalupe River	Guadalupe	Texas	28.822267	-97.016892	V	This study
OQ656517	BAH133	Guadalupe River	Guadalupe	Texas	28.822267	-97.016892	V	This study
OQ656518	BAH134	Guadalupe River	Guadalupe	Texas	28.822267	-97.016892	V	This study
OQ656519	BAH137	Guadalupe River	Guadalupe	Texas	28.822267	-97.016892	V	This study
OQ656520	BAH160	Guadalupe River	Guadalupe	Texas	29.488248	-97.439886	V	This study
OQ656521	BAH162	Guadalupe River	Guadalupe	Texas	29.488248	-97.439886	V	This study
OQ656522	BAH163	Guadalupe River	Guadalupe	Texas	29.488248	-97.439886	V	This study
OQ656523	BAH246	San Saba River	Colorado	Texas	31.21329	-98.71937	IV	This study
OQ656524	BAH247	San Saba River	Colorado	Texas	31.21329	-98.71937	IV	This study
OQ656525	BAH251	San Saba River	Colorado	Texas	31.21329	-98.71937	IV	This study
OQ656526	BAH253	San Saba River	Colorado	Texas	31.21329	-98.71937	IV	This study
OQ656527	BAH254	San Saba River	Colorado	Texas	31.21329	-98.71937	IV	This study
OQ656528	BAH255	San Saba River	Colorado	Texas	31.21329	-98.71937	IV	This study

OQ656529	BAH257	San Saba River	Colorado	Texas	31.21329	-98.71937	IV	This study
OQ656530	BAH258	San Saba River	Colorado	Texas	31.21329	-98.71937	IV	This study
OQ656531	BAH259	San Saba River	Colorado	Texas	31.21329	-98.71937	IV	This study
OQ656532	BAH261	San Saba River	Colorado	Texas	31.22308	-98.78452	IV	This study
OQ656533	BAH262	San Saba River	Colorado	Texas	31.22308	-98.78452	IV	This study
OQ656534	BAH263	San Saba River	Colorado	Texas	31.22308	-98.78452	IV	This study
OQ656535	BAH264	San Saba River	Colorado	Texas	31.22308	-98.78452	IV	This study
OQ656536	BAH265	San Saba River	Colorado	Texas	31.22308	-98.78452	IV	This study
OQ656537	BAH267	San Saba River	Colorado	Texas	31.22308	-98.78452	IV	This study
OQ656538	BAH269	San Saba River	Colorado	Texas	31.22308	-98.78452	IV	This study
OQ656539	BAH270	San Saba River	Colorado	Texas	31.22308	-98.78452	IV	This study
OQ656540	BAH271	San Saba River	Colorado	Texas	31.22308	-98.78452	IV	This study
OQ656541	BAH273	San Saba River	Colorado	Texas	31.22308	-98.78452	IV	This study
OQ656542	BAH290	San Saba River	Colorado	Texas	31.21329	-98.71937	IV	This study
OQ656543	BAH291	San Saba River	Colorado	Texas	31.21329	-98.71937	IV	This study
OQ656544	BAH292	San Saba River	Colorado	Texas	31.21329	-98.71937	IV	This study
OQ656545	BAH294	San Saba River	Colorado	Texas	31.21329	-98.71937	IV	This study
OQ656546	BAH295	San Saba River	Colorado	Texas	31.21329	-98.71937	IV	This study
OQ656547	BAH297	San Saba River	Colorado	Texas	31.21329	-98.71937	IV	This study
OQ656548	BAH298	San Saba River	Colorado	Texas	31.21329	-98.71937	IV	This study
OQ656549	BAH299	San Saba River	Colorado	Texas	31.21329	-98.71937	IV	This study
OQ656550	BAH300	San Saba River	Colorado	Texas	31.21329	-98.71937	IV	This study
OQ656551	BAH301	San Saba River	Colorado	Texas	31.21329	-98.71937	IV	This study
OQ656552	BAH304	San Saba River	Colorado	Texas	31.21329	-98.71937	IV	This study
OQ656553	BAH321	Village Creek	Neches	Texas	30.258898	-94.168618	III	This study
OQ656554	BAH322	Village Creek	Neches	Texas	30.258898	-94.168618	III	This study
OQ656555	BAH323	Village Creek	Neches	Texas	30.258898	-94.168618	III	This study
OQ656556	BAH324	Village Creek	Neches	Texas	30.258898	-94.168618	III	This study
OQ656557	BAH325	Village Creek	Neches	Texas	30.258898	-94.168618	III	This study
OQ656558	BAH326	Village Creek	Neches	Texas	30.258898	-94.168618	III	This study
OQ656559	BAH328	Village Creek	Neches	Texas	30.258898	-94.168618	III	This study
OQ656560	BAH329	Village Creek	Neches	Texas	30.258898	-94.168618	III	This study
OQ656561	BAH332	Village Creek	Neches	Texas	30.258898	-94.168618	III	This study
OQ656562	BAH333	Village Creek	Neches	Texas	30.258898	-94.168618	III	This study
OQ656563	BAH351	Guadalupe River	Guadalupe	Texas	29.150067	-97.316154	V	This study
FJ601220	PP1	St. Croix	Upper Mississippi	Wisconsin	45.3669722	-92.69475	I	Szumowski et al. 2012
FJ601221	PP2	St. Croix	Upper Mississippi	Wisconsin	45.3669722	-92.69475	I	Szumowski et al. 2012
FJ601222	PP3	St. Croix	Upper Mississippi	Wisconsin	45.3553889	-92.703889	I	Szumowski et al. 2012
FJ601223	PP4	St. Croix	Upper Mississippi	Wisconsin	45.3669722	-92.69475	I	Szumowski et al. 2012
FJ601224	PP5	St. Croix	Upper Mississippi	Wisconsin	45.3669722	-92.69475	I	Szumowski et al. 2012
FJ601225	PP6	St. Croix	Upper Mississippi	Wisconsin	45.3669722	-92.69475	I	Szumowski et al. 2012
FJ601226	PP7	St. Croix	Upper Mississippi	Wisconsin	45.3669722	-92.69475	I	Szumowski et al. 2012
FJ601227	PP8	St. Croix	Upper Mississippi	Wisconsin	45.3669722	-92.69475	I	Szumowski et al. 2012
FJ601228	PP9	St. Croix	Upper Mississippi	Wisconsin	45.3669722	-92.69475	I	Szumowski et al. 2012
FJ601229	PP10	St. Croix	Upper Mississippi	Wisconsin	45.3669722	-92.69475	I	Szumowski et al. 2012
FJ601230	PP11	St. Croix	Upper Mississippi	Wisconsin	45.3669722	-92.711417	I	Szumowski et al. 2012
FJ601231	PP12	St. Croix	Upper Mississippi	Wisconsin	45.3669722	-92.69475	I	Szumowski et al. 2012
FJ601233	PP14	St. Croix	Upper Mississippi	Wisconsin	45.3669722	-92.69475	I	Szumowski et al. 2012
FJ601234	PP15	St. Croix	Upper Mississippi	Wisconsin	45.3669722	-92.69475	I	Szumowski et al. 2012
FJ601235	PP16	St. Croix	Upper Mississippi	Wisconsin	45.3669722	-92.69475	I	Szumowski et al. 2012
FJ601236	PP17	St. Croix	Upper Mississippi	Wisconsin	45.3669722	-92.69475	I	Szumowski et al. 2012
FJ601237	PP18	St. Croix	Upper Mississippi	Wisconsin	45.3669722	-92.711417	I	Szumowski et al. 2012
FJ601238	PP19	St. Croix	Upper Mississippi	Wisconsin	45.3669722	-92.69475	I	Szumowski et al. 2012
FJ601239	PP20	St. Croix	Upper Mississippi	Wisconsin	45.3669722	-92.711417	I	Szumowski et al. 2012
FJ601240	PP21	St. Croix	Upper Mississippi	Wisconsin	45.8255278	-92.831667	I	Szumowski et al. 2012
FJ601241	PP22	St. Croix	Upper Mississippi	Wisconsin	45.3553889	-92.703889	I	Szumowski et al. 2012
FJ601242	SIP6	St. Croix	Upper Mississippi	Wisconsin	45.8255278	-92.831667	I	Szumowski et al. 2012
FJ601243	SIP8	St. Croix	Upper Mississippi	Wisconsin	45.8255278	-92.831667	I	Szumowski et al. 2012
FJ601244	SIP9	St. Croix	Upper Mississippi	Wisconsin	45.8255278	-92.831667	I	Szumowski et al. 2012
FJ601245	SIP11	St. Croix	Upper Mississippi	Wisconsin	45.8255278	-92.831667	I	Szumowski et al. 2012
FJ601246	SIP12	St. Croix	Upper Mississippi	Wisconsin	45.8255278	-92.831667	I	Szumowski et al. 2012
FJ601247	SIP13	St. Croix	Upper Mississippi	Wisconsin	45.8255278	-92.831667	I	Szumowski et al. 2012
FJ601248	SIP14	St. Croix	Upper Mississippi	Wisconsin	45.8255278	-92.831667	I	Szumowski et al. 2012
FJ601249	SIP17	St. Croix	Upper Mississippi	Wisconsin	45.8255278	-92.831667	I	Szumowski et al. 2012
FJ601250	SIP18	St. Croix	Upper Mississippi	Wisconsin	45.8255278	-92.831667	I	Szumowski et al. 2012
FJ601251	SIP19	St. Croix	Upper Mississippi	Wisconsin	45.8255278	-92.831667	I	Szumowski et al. 2012
FJ601252	SIP20	St. Croix	Upper Mississippi	Wisconsin	45.8255278	-92.831667	I	Szumowski et al. 2012
FJ601253	HP1	St. Croix	Upper Mississippi	Wisconsin	44.9754722	-92.770556	I	Szumowski et al. 2012
FJ601254	HP2	St. Croix	Upper Mississippi	Wisconsin	44.9754722	-92.770556	I	Szumowski et al. 2012
FJ601255	HP4	St. Croix	Upper Mississippi	Wisconsin	44.9754722	-92.770556	I	Szumowski et al. 2012
FJ601256	HP5	St. Croix	Upper Mississippi	Wisconsin	44.9754722	-92.770556	I	Szumowski et al. 2012
FJ601259	HP12	St. Croix	Upper Mississippi	Wisconsin	44.9754722	-92.770556	I	Szumowski et al. 2012
FJ601260	HP13	St. Croix	Upper Mississippi	Wisconsin	44.9754722	-92.770556	I	Szumowski et al. 2012
FJ601261	HP14	St. Croix	Upper Mississippi	Wisconsin	44.9754722	-92.770556	I	Szumowski et al. 2012
FJ601262	HP15	St. Croix	Upper Mississippi	Wisconsin	44.9754722	-92.770556	I	Szumowski et al. 2012
FJ601263	HP16	St. Croix	Upper Mississippi	Wisconsin	44.9754722	-92.770556	I	Szumowski et al. 2012
FJ601264	HP17	St. Croix	Upper Mississippi	Wisconsin	44.9754722	-92.770556	I	Szumowski et al. 2012
FJ601266	HP20	St. Croix	Upper Mississippi	Wisconsin	44.9754722	-92.770556	I	Szumowski et al. 2012
FJ601267	WRP1	St. Croix	Upper Mississippi	Wisconsin	45.5227222	-92.729	I	Szumowski et al. 2012
FJ601269	WRP11	St. Croix	Upper Mississippi	Wisconsin	45.5227222	-92.729	I	Szumowski et al. 2012
FJ601270	WRP12	St. Croix	Upper Mississippi	Wisconsin	45.5227222	-92.729	I	Szumowski et al. 2012
FJ601271	WRP13	St. Croix	Upper Mississippi	Wisconsin	45.5227222	-92.729	I	Szumowski et al. 2012
FJ601272	WRP14	St. Croix	Upper Mississippi	Wisconsin	45.5227222	-92.729	I	Szumowski et al. 2012
FJ601273	WRP15	St. Croix	Upper Mississippi	Wisconsin	45.5227222	-92.729	I	Szumowski et al. 2012
FJ601274	WRP16	St. Croix	Upper Mississippi	Wisconsin	45.5227222	-92.729	I	Szumowski et al. 2012
FJ601275	WRP17	St. Croix	Upper Mississippi	Wisconsin	45.5227222	-92.729	I	Szumowski et al. 2012

FJ601276	WRP18	St. Croix	Upper Mississippi	Wisconsin	45.5227222	-92.729	I	Szumowski et al. 2012
FJ601277	WRP19	St. Croix	Upper Mississippi	Wisconsin	45.5227222	-92.729	I	Szumowski et al. 2012
FJ601278	WRP20	St. Croix	Upper Mississippi	Wisconsin	45.5227222	-92.729	I	Szumowski et al. 2012
FJ601279	Q1MCOXA20	St. Croix	Upper Mississippi	Wisconsin	45.5227222	-92.729	I	Szumowski et al. 2012
FJ601280	Q22MCOXA19	St. Croix	Upper Mississippi	Wisconsin	45.5227222	-92.729	I	Szumowski et al. 2012
OQ656564	GRQP01	Grand	Great Lakes	Ontario	42.98532	-79.872115	I	This study
OQ656565	GRQP02	Grand	Great Lakes	Ontario	42.98532	-79.872115	I	This study
OQ656566	GRQP03	Grand	Great Lakes	Ontario	42.98532	-79.872115	I	This study
OQ656567	GRQP04	Grand	Great Lakes	Ontario	42.98532	-79.872115	I	This study
OQ656568	GRQP05	Grand	Great Lakes	Ontario	42.98532	-79.872115	I	This study
OQ656569	GRQP06	Grand	Great Lakes	Ontario	42.98532	-79.872115	I	This study
OQ656570	GRQP07	Grand	Great Lakes	Ontario	42.98532	-79.872115	I	This study
OQ656571	GRQP08	Grand	Great Lakes	Ontario	42.98532	-79.872115	I	This study
OQ656572	GRQP09	Grand	Great Lakes	Ontario	42.98532	-79.872115	I	This study
OQ656573	GRQP10	Grand	Great Lakes	Ontario	42.98532	-79.872115	I	This study
OQ656574	GRQP12	Grand	Great Lakes	Ontario	42.98532	-79.872115	I	This study
OQ656575	GRQP13	Grand	Great Lakes	Ontario	42.98532	-79.872115	I	This study
OQ656576	GRQP14	Grand	Great Lakes	Ontario	42.98532	-79.872115	I	This study
OQ656577	GRQP15	Grand	Great Lakes	Ontario	42.98532	-79.872115	I	This study
OQ656578	GRQP16	Grand	Great Lakes	Ontario	42.98532	-79.872115	I	This study
OQ656579	GRQP17	Grand	Great Lakes	Ontario	42.98532	-79.872115	I	This study
OQ656580	GRQP18	Grand	Great Lakes	Ontario	42.98532	-79.872115	I	This study
OQ656581	GRQP19	Grand	Great Lakes	Ontario	42.98532	-79.872115	I	This study
OQ656582	GRQP20	Grand	Great Lakes	Ontario	42.98532	-79.872115	I	This study
OQ656583	GUADL1	Guadalupe River	Guadalupe	Texas	28.822267	-97.016892	V	This study
OQ656584	GUADL10	Guadalupe River	Guadalupe	Texas	28.822267	-97.016892	V	This study
OQ656585	GUADL12	Guadalupe River	Guadalupe	Texas	28.822267	-97.016892	V	This study
OQ656586	GUADL2	Guadalupe River	Guadalupe	Texas	28.822267	-97.016892	V	This study
OQ656587	GUADL3	Guadalupe River	Guadalupe	Texas	28.822267	-97.016892	V	This study
OQ656588	GUADL6	Guadalupe River	Guadalupe	Texas	28.822267	-97.016892	V	This study
OQ656589	GUADL7	Guadalupe River	Guadalupe	Texas	28.822267	-97.016892	V	This study
OQ656590	GUADL9	Guadalupe River	Guadalupe	Texas	28.822267	-97.016892	V	This study
HM852934	BM20258	St. Croix	Upper Mississippi	Wisconsin	NA	NA	I	Boyer et al. 2011
HM852935	BM20241	St. Croix	Upper Mississippi	Wisconsin	NA	NA	I	Boyer et al. 2011
MG020451	NA	Sulphur River	Red	Texas	33.36	-94.8	I	Marshall et al. 2018
MH361842	QaurGua001	San Antonio River	Guadalupe	Texas	28.531484	-97.042921	V	Johnson et al. 2018
MH361843	QaurGua004	Guadalupe River	Guadalupe	Texas	28.83164	-97.05937	V	Johnson et al. 2018
MH361844	QaurGua006	Guadalupe River	Guadalupe	Texas	28.83164	-97.05937	V	Johnson et al. 2018
MH361845	QaurGua008	Guadalupe River	Guadalupe	Texas	28.83164	-97.05937	V	Johnson et al. 2018
MH361846	QaurGua009	Guadalupe River	Guadalupe	Texas	28.83164	-97.05937	V	Johnson et al. 2018
MH361847	QaurGua012	Guadalupe River	Guadalupe	Texas	29.67078	-97.69561	V	Johnson et al. 2018
MH361848	QaurGua013	Guadalupe River	Guadalupe	Texas	29.67078	-97.69561	V	Johnson et al. 2018
MH361849	QaurGua014	Guadalupe River	Guadalupe	Texas	29.67078	-97.69561	V	Johnson et al. 2018
MH361850	QaurGua015	Guadalupe River	Guadalupe	Texas	29.67078	-97.69561	V	Johnson et al. 2018
MH361851	QaurGua016	Guadalupe River	Guadalupe	Texas	29.67078	-97.69561	V	Johnson et al. 2018
MH361852	QaurGua017	Guadalupe River	Guadalupe	Texas	29.67078	-97.69561	V	Johnson et al. 2018
MH361853	QaurGua018	Guadalupe River	Guadalupe	Texas	29.67078	-97.69561	V	Johnson et al. 2018
MH361854	QaurGua021	San Antonio River	Guadalupe	Texas	28.651169	-97.432994	V	Johnson et al. 2018
MH361855	QaurNue027	Frio	San Antonio	Texas	28.46104	-98.19073	V	Johnson et al. 2018
MH361856	QaurNue028	Frio	Nueces	Texas	28.46104	-98.19073	V	Johnson et al. 2018
MH361857	QaurNue031	Nueces	Nueces	Texas	28.37436	-98.11561	V	Johnson et al. 2018
MH361858	QaurGua040	Guadalupe River	Guadalupe	Texas	29.221294	-97.329695	V	Johnson et al. 2018
MH361859	QaurGua041	Guadalupe River	Guadalupe	Texas	29.221294	-97.329695	V	Johnson et al. 2018
MH361860	QaurGua042	Guadalupe River	Guadalupe	Texas	29.221294	-97.329695	V	Johnson et al. 2018
MH361861	QaurGua043	Guadalupe River	Guadalupe	Texas	29.221294	-97.329695	V	Johnson et al. 2018
MH361862	QaurGua044	Guadalupe River	Guadalupe	Texas	29.221294	-97.329695	V	Johnson et al. 2018
MH361863	QaurGua045	Guadalupe River	Guadalupe	Texas	29.221294	-97.329695	V	Johnson et al. 2018
MH361864	QaurGua046	Guadalupe River	Guadalupe	Texas	29.221294	-97.329695	V	Johnson et al. 2018
MH361865	QaurGua047	Guadalupe River	Guadalupe	Texas	29.221294	-97.329695	V	Johnson et al. 2018
MH361866	QaurGua048	Guadalupe River	Guadalupe	Texas	29.221294	-97.329695	V	Johnson et al. 2018
MH361867	QaurGua049	Guadalupe River	Guadalupe	Texas	29.221294	-97.329695	V	Johnson et al. 2018
MH361868	QaurNue050	Nueces	Nueces	Texas	28.48684	-98.23867	V	Johnson et al. 2018
MH361869	QaurNue051	Nueces	Nueces	Texas	28.48684	-98.23867	V	Johnson et al. 2018
MH361870	QaurNue052	Nueces	Nueces	Texas	28.48684	-98.23867	V	Johnson et al. 2018
MH361871	QaurNue054	Nueces	Nueces	Texas	28.48684	-98.23867	V	Johnson et al. 2018
MH361872	QaurNue057	Nueces	Nueces	Texas	28.48684	-98.23867	V	Johnson et al. 2018
MH361873	QaurNue058	Nueces	Nueces	Texas	28.48684	-98.23867	V	Johnson et al. 2018
MH361874	QaurNue059	Nueces	Nueces	Texas	28.48684	-98.23867	V	Johnson et al. 2018
MH361875	QaurNue060	Nueces	Nueces	Texas	28.48684	-98.23867	V	Johnson et al. 2018
MH361876	QaurNue061	Nueces	Nueces	Texas	28.48684	-98.23867	V	Johnson et al. 2018
MH361877	QaurNue062	Nueces	Nueces	Texas	28.337458	-98.086636	V	Johnson et al. 2018
MH361878	QaurNue063	Nueces	Nueces	Texas	28.337458	-98.086636	V	Johnson et al. 2018
MH361879	QaurNue064	Nueces	Nueces	Texas	28.337458	-98.086636	V	Johnson et al. 2018
MH361880	QaurNue065	Nueces	Nueces	Texas	28.337458	-98.086636	V	Johnson et al. 2018
MH361881	QaurNue066	Nueces	Nueces	Texas	28.337458	-98.086636	V	Johnson et al. 2018
MH361882	QaurNue067	Nueces	Nueces	Texas	28.337458	-98.086636	V	Johnson et al. 2018
MH361883	QaurNue068	Nueces	Nueces	Texas	28.337458	-98.086636	V	Johnson et al. 2018
MH361884	QaurNue069	Nueces	Nueces	Texas	28.337458	-98.086636	V	Johnson et al. 2018
MH361885	QaurNue070	Nueces	Nueces	Texas	28.337458	-98.086636	V	Johnson et al. 2018
MH361886	QaurNue071	Nueces	Nueces	Texas	28.337458	-98.086636	V	Johnson et al. 2018
MH361887	QaurNue072	Nueces	Nueces	Texas	28.337458	-98.086636	V	Johnson et al. 2018
MH361888	QaurNue073	Nueces	Nueces	Texas	28.337458	-98.086636	V	Johnson et al. 2018
MH361889	QaurNue074	Nueces	Nueces	Texas	28.337458	-98.086636	V	Johnson et al. 2018
MH361890	QaurNue075	Nueces	Nueces	Texas	28.337458	-98.086636	V	Johnson et al. 2018
MH361891	QaurNue076	Nueces	Nueces	Texas	28.337458	-98.086636	V	Johnson et al. 2018
MH361892	QaurNue077	Nueces	Nueces	Texas	28.337458	-98.086636	V	Johnson et al. 2018

MH361893	QaurNue078	Nueces	Nueces	Texas	28.337458	-98.086636	V	Johnson et al. 2018
MH361894	QaurNue079	Nueces	Nueces	Texas	28.337458	-98.086636	V	Johnson et al. 2018
MH361895	QaurNue080	Nueces	Nueces	Texas	28.382964	-98.109617	V	Johnson et al. 2018
MH361896	QaurNue081	Nueces	Nueces	Texas	28.382964	-98.109617	V	Johnson et al. 2018
MH361897	QaurNue082	Nueces	Nueces	Texas	28.382964	-98.109617	V	Johnson et al. 2018
MH361898	QaurNue083	Nueces	Nueces	Texas	28.382964	-98.109617	V	Johnson et al. 2018
MH361899	QaurNue085	Nueces	Nueces	Texas	28.382964	-98.109617	V	Johnson et al. 2018
MH361900	QaurNue086	Nueces	Nueces	Texas	28.382964	-98.109617	V	Johnson et al. 2018
MH361901	QaurNue088	Nueces	Nueces	Texas	28.382964	-98.109617	V	Johnson et al. 2018
MH361902	QaurNue089	Nueces	Nueces	Texas	28.382964	-98.109617	V	Johnson et al. 2018
MH361903	QaurGua090	Guadalupe River	Guadalupe	Texas	29.56274	-97.543455	V	Johnson et al. 2018
MH361904	QaurGua091	Guadalupe River	Guadalupe	Texas	29.56274	-97.543455	V	Johnson et al. 2018
MH361905	QaurGua092	Guadalupe River	Guadalupe	Texas	29.56274	-97.543455	V	Johnson et al. 2018
MH361906	QaurGua093	Guadalupe River	Guadalupe	Texas	29.56274	-97.543455	V	Johnson et al. 2018
MH361907	QaurGua094	Guadalupe River	Guadalupe	Texas	29.56274	-97.543455	V	Johnson et al. 2018
MH361908	QaurGua095	Guadalupe River	Guadalupe	Texas	29.56274	-97.543455	V	Johnson et al. 2018
MH361909	QaurGua096	Guadalupe River	Guadalupe	Texas	29.56274	-97.543455	V	Johnson et al. 2018
MH361910	QaurNec112	Nueces	Nueces	Texas	NA	NA	V	Johnson et al. 2018
MH361911	QhouCol001	Colorado River	Colorado	Texas	29.45354	-96.39587	IV	Johnson et al. 2018
MH361912	QhouCol003	Colorado River	Colorado	Texas	29.45354	-96.39587	IV	Johnson et al. 2018
MH361913	QhouCol004	San Saba River	Colorado	Texas	31.20738	-98.68761	IV	Johnson et al. 2018
MH361914	QhouCol005	San Saba River	Colorado	Texas	31.20738	-98.68761	IV	Johnson et al. 2018
MH361915	QhouCol006	San Saba River	Colorado	Texas	31.20738	-98.68761	IV	Johnson et al. 2018
MH361916	QhouCol007	San Saba River	Colorado	Texas	31.20738	-98.68761	IV	Johnson et al. 2018
MH361917	QhouCol009	San Saba River	Colorado	Texas	31.20738	-98.68761	IV	Johnson et al. 2018
MH361918	QhouBra011	Brazos	Brazos	Texas	30.8676502	-96.695637	IV	Johnson et al. 2018
MH361919	QhouBra012	Brazos	Brazos	Texas	30.8676502	-96.695637	IV	Johnson et al. 2018
MH361920	QhouBra013	Brazos	Brazos	Texas	30.8676502	-96.695637	IV	Johnson et al. 2018
MH361921	QhouBra014	Brazos	Brazos	Texas	30.8676502	-96.695637	IV	Johnson et al. 2018
MH361922	QhouBra015	Brazos	Brazos	Texas	30.8676502	-96.695637	IV	Johnson et al. 2018
MH361923	QhouBra017	Brazos	Brazos	Texas	31.9574037	-98.458971	IV	Johnson et al. 2018
MH361924	QpetCol026	San Saba River	Colorado	Texas	31.212419	-98.79837	IV	Johnson et al. 2018
MH361925	QhouBra019	Brazos	Brazos	Texas	31.34928	-97.60237	IV	Johnson et al. 2018
MH361926	QhouBra020	Brazos	Brazos	Texas	31.34928	-97.60237	IV	Johnson et al. 2018
MH361927	QhouBra021	Brazos	Brazos	Texas	31.34928	-97.60237	IV	Johnson et al. 2018
MH361928	QhouBra023	Brazos	Brazos	Texas	31.34928	-97.60237	IV	Johnson et al. 2018
MH361929	QhouCol029	Colorado River	Colorado	Texas	31.2617789	-98.594768	IV	Johnson et al. 2018
MH361930	QhouBra035	Brazos	Brazos	Texas	30.5811176	-96.441506	IV	Johnson et al. 2018
MH361931	QhouBra036	Brazos	Brazos	Texas	30.5811176	-96.441506	IV	Johnson et al. 2018
MH361932	QhouBra037	Brazos	Brazos	Texas	30.5811176	-96.441506	IV	Johnson et al. 2018
MH361933	QhouBra038	Brazos	Brazos	Texas	30.5811176	-96.441506	IV	Johnson et al. 2018
MH361934	QhouBra041	Brazos	Brazos	Texas	30.695341	-97.27804	IV	Johnson et al. 2018
MH361935	QhouBra042	Brazos	Brazos	Texas	30.695341	-97.27804	IV	Johnson et al. 2018
MH361936	QpetCol079	Llano River	Colorado	Texas	30.658673	-99.3245	IV	Johnson et al. 2018
MH361937	QhouBra044	Brazos	Brazos	Texas	30.81558	-97.13651	IV	Johnson et al. 2018
MH361938	QhouBra047	Brazos	Brazos	Texas	30.81558	-97.13651	IV	Johnson et al. 2018
MH361939	QmorCol060	Colorado River	Colorado	Texas	29.173116	-96.067204	IV	Johnson et al. 2018
MH361940	QmorCol061	Colorado River	Colorado	Texas	29.173116	-96.067204	IV	Johnson et al. 2018
MH361941	QmorCol062	Colorado River	Colorado	Texas	29.173116	-96.067204	IV	Johnson et al. 2018
MH361942	QmorCol063	Colorado River	Colorado	Texas	29.173116	-96.067204	IV	Johnson et al. 2018
MH361943	QmorSab001	Sabine	Sabine	Texas	30.6652604	-93.658055	III	Johnson et al. 2018
MH361944	QmorSab004	Trinity	Trinity	Texas	32.592543	-96.486377	III	Johnson et al. 2018
MH361945	QmorSab005	Sabine	Sabine	Texas	30.3565919	-93.760699	III	Johnson et al. 2018
MH361946	QmorSab006	Sabine	Sabine	Texas	31.0163485	-93.365165	III	Johnson et al. 2018
MH361947	QmorSab008	Sabine	Sabine	Texas	32.5573098	-95.205639	III	Johnson et al. 2018
MH361948	QmorTri013	Trinity	Trinity	Texas	31.3352	-95.65641	III	Johnson et al. 2018
MH361949	QmorTri014	Trinity	Trinity	Texas	31.64941	-95.7897	III	Johnson et al. 2018
MH361950	QmorTri017	Sabine	Sabine	Texas	30.591914	-93.698572	III	Johnson et al. 2018
MH361951	QmorTri018	Sabine	Sabine	Texas	30.591914	-93.698572	III	Johnson et al. 2018
MH361952	QmorNec019	Neches	Neches	Texas	31.885207	-95.431684	III	Johnson et al. 2018
MH361953	QmorNec020	Neches	Neches	Texas	31.885207	-95.431684	III	Johnson et al. 2018
MH361954	QmorNec021	Neches	Neches	Texas	31.885207	-95.431684	III	Johnson et al. 2018
MH361955	QmorNec022	Neches	Neches	Texas	31.885207	-95.431684	III	Johnson et al. 2018
MH361956	QmorNec023	Neches	Neches	Texas	31.885207	-95.431684	III	Johnson et al. 2018
MH361957	QmorNec024	Neches	Neches	Texas	31.885207	-95.431684	III	Johnson et al. 2018
MH361958	QmorNec025	Neches	Neches	Texas	31.885207	-95.431684	III	Johnson et al. 2018
MH361959	QmorNec026	Neches	Neches	Texas	31.885207	-95.431684	III	Johnson et al. 2018
MH361960	QmorNec028	Neches	Neches	Texas	31.885207	-95.431684	III	Johnson et al. 2018
MH361961	QmorNec029	Neches	Neches	Texas	31.885207	-95.431684	III	Johnson et al. 2018
MH361962	QmorNec030	Neches	Neches	Texas	31.885207	-95.431684	III	Johnson et al. 2018
MH361963	QmorNec031	Neches	Neches	Texas	31.945598	-95.444602	III	Johnson et al. 2018
MH361964	QmorNec032	Neches	Neches	Texas	31.945598	-95.444602	III	Johnson et al. 2018
MH361965	QmorNec033	Neches	Neches	Texas	31.945598	-95.444602	III	Johnson et al. 2018
MH361966	QmorNec034	Neches	Neches	Texas	31.945598	-95.444602	III	Johnson et al. 2018
MH361967	QmorNec035	Neches	Neches	Texas	31.945598	-95.444602	III	Johnson et al. 2018
MH361968	QmorNec036	Neches	Neches	Texas	31.945598	-95.444602	III	Johnson et al. 2018
MH361969	QmorNec037	Neches	Neches	Texas	31.945598	-95.444602	III	Johnson et al. 2018
MH361970	QmorNec038	Neches	Neches	Texas	31.945598	-95.444602	III	Johnson et al. 2018
MH361971	QmorNec039	Neches	Neches	Texas	31.945598	-95.444602	III	Johnson et al. 2018
MH361972	QmorNec040	Neches	Neches	Texas	31.945598	-95.444602	III	Johnson et al. 2018
MH361973	QmorNec041	Neches	Neches	Texas	31.945598	-95.444602	III	Johnson et al. 2018
MH361974	QmorNec042	Neches	Neches	Texas	31.945598	-95.444602	III	Johnson et al. 2018
MH361975	QmorTri043	Grapevine Lake	Trinity	Texas	32.980521	-97.09841	III	Johnson et al. 2018
MH361976	QmorTri044	Grapevine Lake	Trinity	Texas	32.980521	-97.09841	III	Johnson et al. 2018
MH361977	QmorTri046	Grapevine Lake	Trinity	Texas	32.980521	-97.09841	III	Johnson et al. 2018
MH361978	QmorTri047	Grapevine Lake	Trinity	Texas	32.980521	-97.09841	III	Johnson et al. 2018

MH361979	QmorTri048	Grapevine Lake	Trinity	Texas	33.067064	-96.963914	III	Johnson et al. 2018
MH361980	QmorTri049	Trinity	Trinity	Texas	33.065862	-96.964305	III	Johnson et al. 2018
MH361981	QmorRed051	Red River	Red	Texas	32.739928	-94.441666	I	Johnson et al. 2018
MH361982	QmorRed052	Red River	Red	Texas	32.739928	-94.441666	I	Johnson et al. 2018
MH361983	QmorRed053	Red River	Red	Texas	32.739928	-94.441666	I	Johnson et al. 2018
MH361984	QmorRed054	Red River	Red	Texas	32.739928	-94.441666	I	Johnson et al. 2018
MH361985	QmorRed055	Red River	Red	Texas	32.739928	-94.441666	I	Johnson et al. 2018
MH361986	QmorTri055	Trinity	Trinity	Texas	31.683591	-95.792124	III	Johnson et al. 2018
MH361987	QmorTri056	Trinity	Trinity	Texas	31.683591	-95.792124	III	Johnson et al. 2018
MH361988	QmorTri057	Trinity	Trinity	Texas	31.683591	-95.792124	III	Johnson et al. 2018
MH361989	QmorTri058	Trinity	Trinity	Texas	31.683591	-95.792124	III	Johnson et al. 2018
MH361990	QmorTri059	Trinity	Trinity	Texas	31.683591	-95.792124	III	Johnson et al. 2018
MH361991	QmorSab066	Sabine	Sabine	Texas	32.65716	-95.3615	III	Johnson et al. 2018
MH361992	QmorSab068	Sabine	Sabine	Texas	32.65716	-95.3615	III	Johnson et al. 2018
MH361993	QmorSaJ069	San Jacinto River	San Jacinto	Texas	30.134462	-95.59674	III	Johnson et al. 2018
MH361994	QmorSaJ070	San Jacinto River	San Jacinto	Texas	30.210398	-95.397995	III	Johnson et al. 2018
MH361995	QmorSaJ071	San Jacinto River	San Jacinto	Texas	30.29125	-95.121296	III	Johnson et al. 2018
MH361996	QmorSaJ072	San Jacinto River	San Jacinto	Texas	30.342505	-95.542981	III	Johnson et al. 2018
MH361997	QmorSaJ073	San Jacinto River	San Jacinto	Texas	30.342505	-95.542981	III	Johnson et al. 2018
MH361998	QmorNec087	Neches	Neches	Texas	31.19669	-94.85869	III	Johnson et al. 2018
MH361999	QmorNec088	Neches	Neches	Texas	31.19669	-94.85869	III	Johnson et al. 2018
MH362000	QmorNec089	Neches	Neches	Texas	31.19669	-94.85869	III	Johnson et al. 2018
MH362001	QmorSaJ091	San Jacinto River	San Jacinto	Texas	30.42912	-95.12973	III	Johnson et al. 2018
MH362002	QmorSaJ092	San Jacinto River	San Jacinto	Texas	30.42912	-95.12973	III	Johnson et al. 2018
MH362003	QmorSaJ093	San Jacinto River	San Jacinto	Texas	30.42912	-95.12973	III	Johnson et al. 2018
MH362004	QmorSaJ094	San Jacinto River	San Jacinto	Texas	30.42912	-95.12973	III	Johnson et al. 2018
MH362005	QpusRed001	Red River	Red	Texas	33.854656	-95.341305	I	Johnson et al. 2018
MH362006	QpusRed002	Red River	Red	Texas	33.854656	-95.341305	I	Johnson et al. 2018
MH362007	QpusRed004	Red River	Red	Texas	33.854656	-95.341305	I	Johnson et al. 2018
MH362008	QpusRed005	Red River	Red	Texas	33.854656	-95.341305	I	Johnson et al. 2018
MH362009	QpusRed006	Red River	Red	Texas	33.854656	-95.341305	I	Johnson et al. 2018
MH362010	QpusRed007	Red River	Red	Texas	33.854656	-95.341305	I	Johnson et al. 2018
MH362011	QpusRed008	Red River	Red	Texas	33.854656	-95.341305	I	Johnson et al. 2018
MH362012	QpusRed009	Red River	Red	Texas	33.854656	-95.341305	I	Johnson et al. 2018
MH362013	QpusRed010	Red River	Red	Texas	33.854656	-95.341305	I	Johnson et al. 2018
MH362014	QpusOhi046	Ohio River	Ohio	Ohio	NA	NA	I	Johnson et al. 2018
MH362015	QpusOhi047	Ohio River	Ohio	Ohio	NA	NA	I	Johnson et al. 2018
MH362016	QpusStC048	St. Croix	Upper Mississippi	Wisconsin	NA	NA	I	Johnson et al. 2018
MH362017	QpusRed049	Red River	Red	Texas	NA	NA	I	Johnson et al. 2018
MH362018	QnodRed004	Red River	Red	Texas	NA	NA	I	Johnson et al. 2018
MH362019	QspeTri019	Trinity	Trinity	Texas	NA	NA	III	Johnson et al. 2018
MH362020	QpusOhi050	Ohio River	Ohio	Ohio	NA	NA	I	Johnson et al. 2018
MH362021	QpusOhi051	Ohio River	Ohio	Ohio	39.3912312	-81.420944	I	Johnson et al. 2018
MH362022	QpusOhi052	Ohio River	Ohio	Ohio	39.3912312	-81.420944	I	Johnson et al. 2018
MH362023	QpusOhi053	Ohio River	Ohio	Ohio	39.3912312	-81.420944	I	Johnson et al. 2018
MH362024	QpusOhi054	Ohio River	Ohio	Ohio	39.3912312	-81.420944	I	Johnson et al. 2018
MH362025	QpusOhi055	Ohio River	Ohio	Ohio	39.3912312	-81.420944	I	Johnson et al. 2018
MH362026	QpusOhi056	Ohio River	Ohio	Ohio	39.3912312	-81.420944	I	Johnson et al. 2018
MH362027	QpusRed020	Red River	Red	Texas	33.691899	-93.965148	I	Johnson et al. 2018
MH362028	QpusRed021	Red River	Red	Texas	33.691899	-93.965148	I	Johnson et al. 2018
MH362029	QpusRed022	Red River	Red	Texas	33.691899	-93.965148	I	Johnson et al. 2018
MH362030	QpusRed023	Red River	Red	Texas	33.691899	-93.965148	I	Johnson et al. 2018
MH362031	QpusRed024	Red River	Red	Texas	33.691899	-93.965148	I	Johnson et al. 2018
MH362032	QpusRed025	Red River	Red	Texas	33.691899	-93.965148	I	Johnson et al. 2018
MH362033	QpusRed026	Red River	Red	Texas	33.691899	-93.965148	I	Johnson et al. 2018
MH362034	QpusRed027	Red River	Red	Texas	33.691899	-93.965148	I	Johnson et al. 2018
MH362035	QpusRed028	Red River	Red	Texas	33.691899	-93.965148	I	Johnson et al. 2018
MH362036	QpusRed029	Red River	Red	Texas	33.691899	-93.965148	I	Johnson et al. 2018
MH362037	QpusOua030	Saline River	Lower Mississippi	Arkansas	33.34564	-91.98279	I	Johnson et al. 2018
MH362038	QpusOua031	Saline River	Lower Mississippi	Arkansas	33.34564	-91.98279	I	Johnson et al. 2018
MH362039	QpusOua032	Saline River	Lower Mississippi	Arkansas	33.34564	-91.98279	I	Johnson et al. 2018
MH362040	QpusOua033	Saline River	Lower Mississippi	Arkansas	33.34564	-91.98279	I	Johnson et al. 2018
MH362041	QpusOua034	Saline River	Lower Mississippi	Arkansas	33.34564	-91.98279	I	Johnson et al. 2018
MH362042	QpusOua035	Saline River	Lower Mississippi	Arkansas	33.34564	-91.98279	I	Johnson et al. 2018
MH362043	QpusOua036	Saline River	Lower Mississippi	Arkansas	33.34564	-91.98279	I	Johnson et al. 2018
MH362044	QpusOua037	Saline River	Lower Mississippi	Arkansas	33.34564	-91.98279	I	Johnson et al. 2018
MH362045	QpusOua038	Saline River	Lower Mississippi	Arkansas	33.34564	-91.98279	I	Johnson et al. 2018
MH362046	QpusStF039	St. Francis	Lower Mississippi	Arkansas	35.11766	-90.31891	I	Johnson et al. 2018
MH362047	QpusStF040	St. Francis	Lower Mississippi	Arkansas	35.11766	-90.31891	I	Johnson et al. 2018
MH362048	QpusStF041	St. Francis	Lower Mississippi	Arkansas	35.11766	-90.31891	I	Johnson et al. 2018
MH362049	QpusStF043	St. Francis	Lower Mississippi	Arkansas	35.11766	-90.31891	I	Johnson et al. 2018
MH362050	QpusStF057	St. Francis	Lower Mississippi	Arkansas	35.11766	-90.31891	I	Johnson et al. 2018
MH362051	QpusStF058	St. Francis	Lower Mississippi	Arkansas	35.11766	-90.31891	I	Johnson et al. 2018
MH362052	QpusStF059	St. Francis	Lower Mississippi	Arkansas	35.11766	-90.31891	I	Johnson et al. 2018
MH362054	QpusNeo062	Neosho	Arkansas	Oklahoma	36.568	-94.504	I	Johnson et al. 2018
MH362055	QpusNeo063	Neosho	Arkansas	Oklahoma	36.568	-94.504	I	Johnson et al. 2018
MH362056	QpusNeo064	Neosho	Arkansas	Oklahoma	36.568	-94.504	I	Johnson et al. 2018
MH362057	QpusOsa065	Osage	Missouri	Missouri	37.9815	-93.3081	I	Johnson et al. 2018
MH362058	QpusOsa067	Osage	Missouri	Missouri	37.9815	-93.3081	I	Johnson et al. 2018
MH362059	QpusOsa068	Osage	Missouri	Missouri	37.9815	-93.3081	I	Johnson et al. 2018
MH362060	QpusOsa070	Osage	Missouri	Missouri	37.9815	-93.3081	I	Johnson et al. 2018
MH362061	QpusStF071	St. Francis	Lower Mississippi	Arkansas	37.2371	-90.4857	I	Johnson et al. 2018
MH362062	QpusStF072	St. Francis	Lower Mississippi	Arkansas	37.2371	-90.4857	I	Johnson et al. 2018
MH362063	QpusStF073	St. Francis	Lower Mississippi	Arkansas	37.2371	-90.4857	I	Johnson et al. 2018
MH362064	QpusStF074	St. Francis	Lower Mississippi	Arkansas	37.2371	-90.4857	I	Johnson et al. 2018
MH362065	QpusStC079	St. Croix	Upper Mississippi	Wisconsin	45.3955278	-92.662783	I	Johnson et al. 2018

MH362066	QpusStC080	St. Croix	Upper Mississippi	Wisconsin	45.3955278	-92.662783	I	Johnson et al. 2018
MH362067	QpusStC081	St. Croix	Upper Mississippi	Wisconsin	45.3955278	-92.662783	I	Johnson et al. 2018
MH362068	QpusStC082	St. Croix	Upper Mississippi	Wisconsin	45.3955278	-92.662783	I	Johnson et al. 2018
MH362069	QpusOua084	Ouachita	Lower Mississippi	Louisiana	32.9159	-92.08025	I	Johnson et al. 2018
MH362070	QpusOua085	Ouachita	Lower Mississippi	Louisiana	32.9159	-92.08025	I	Johnson et al. 2018
MH362071	QpusOua086	Ouachita	Lower Mississippi	Louisiana	32.9159	-92.08025	I	Johnson et al. 2018
MH362072	QpusOua087	Ouachita	Lower Mississippi	Louisiana	32.9159	-92.08025	I	Johnson et al. 2018
MH362073	QpusOua088	Ouachita	Lower Mississippi	Louisiana	32.9159	-92.08025	I	Johnson et al. 2018
MH362075	QspeOua024	Ouachita	Lower Mississippi	Louisiana	32.9159	-92.08025	I	Johnson et al. 2018
MH362076	QrefPas001	Pascagoula	South Atlantic-Gulf	Mississippi	30.63228	-88.652401	II	Johnson et al. 2018
MH362077	QrefPas002	Pascagoula	South Atlantic-Gulf	Mississippi	30.63228	-88.652401	II	Johnson et al. 2018
MH362078	QrefPas003	Pascagoula	South Atlantic-Gulf	Mississippi	30.63228	-88.652401	II	Johnson et al. 2018
MH362079	QrefPas004	Pascagoula	South Atlantic-Gulf	Mississippi	30.63228	-88.652401	II	Johnson et al. 2018
MH362080	QrefPas005	Pascagoula	South Atlantic-Gulf	Mississippi	30.63228	-88.652401	II	Johnson et al. 2018
MH362081	QrefPrl006	Bogue Chitto	South Atlantic-Gulf	Louisiana	30.62254	-89.87655	II	Johnson et al. 2018
MH362082	QrefPrl007	Bogue Chitto	South Atlantic-Gulf	Louisiana	30.62254	-89.87655	II	Johnson et al. 2018
MH362083	QrefPrl008	Bogue Chitto	South Atlantic-Gulf	Louisiana	30.62254	-89.87655	II	Johnson et al. 2018
MH362084	QrefPrl009	Bogue Chitto	South Atlantic-Gulf	Louisiana	30.62254	-89.87655	II	Johnson et al. 2018
MH362085	QrefPrl010	Bogue Chitto	South Atlantic-Gulf	Louisiana	30.62254	-89.87655	II	Johnson et al. 2018
MK503296	MK503296	San Marcos	Guadalupe	Texas	NA	NA	V	Lopes-Lima et al. 2019
MK503298	MK503298	San Antonio River	Guadalupe	Texas	NA	NA	V	Lopes-Lima et al. 2019
MK503299	MK503299	San Antonio River	Guadalupe	Texas	NA	NA	V	Lopes-Lima et al. 2019
MK503300	MK503300	San Antonio River	Guadalupe	Texas	NA	NA	V	Lopes-Lima et al. 2019
MK503301	MK503301	San Antonio River	Guadalupe	Texas	NA	NA	V	Lopes-Lima et al. 2019
MK503302	MK503302	Guadalupe River	Guadalupe	Texas	NA	NA	V	Lopes-Lima et al. 2019
MK503303	MK503303	Colorado River	Colorado	Texas	NA	NA	V	Lopes-Lima et al. 2019
MK503304	MK503304	Colorado River	Colorado	Texas	NA	NA	V	Lopes-Lima et al. 2019
MK503305	MK503305	Colorado River	Colorado	Texas	NA	NA	V	Lopes-Lima et al. 2019
MK503306	MK503306	Sandy Creek	Neches	Texas	NA	NA	V	Lopes-Lima et al. 2019
MK503308	MK503308	Village Creek	Neches	Texas	NA	NA	V	Lopes-Lima et al. 2019
MK503314	MK503314	Nueces	Nueces	Texas	NA	NA	V	Lopes-Lima et al. 2019
MK503315	MK503315	Nueces	Nueces	Texas	NA	NA	V	Lopes-Lima et al. 2019
MK503319	MK503319	Guadalupe River	Guadalupe	Texas	NA	NA	V	Lopes-Lima et al. 2019
MK503320	MK503320	Guadalupe River	Guadalupe	Texas	NA	NA	V	Lopes-Lima et al. 2019
MK503322	MK503322	Trinity	Trinity	Texas	NA	NA	III	Lopes-Lima et al. 2019
MK503323	MK503323	Trinity	Trinity	Texas	NA	NA	III	Lopes-Lima et al. 2019
MK503324	MK503324	Trinity	Trinity	Texas	NA	NA	III	Lopes-Lima et al. 2019
OQ656591	SRQP01	Sydenham	Great Lakes	Ontario	42.5892	-82.12808	I	This study
OQ656592	SRQP02	Sydenham	Great Lakes	Ontario	42.5892	-82.12808	I	This study
OQ656593	SRQP03	Sydenham	Great Lakes	Ontario	42.5892	-82.12808	I	This study
OQ656594	SRQP04	Sydenham	Great Lakes	Ontario	42.5892	-82.12808	I	This study
OQ656595	SRQP05	Sydenham	Great Lakes	Ontario	42.5892	-82.12808	I	This study
OQ656596	SRQP07	Sydenham	Great Lakes	Ontario	42.5892	-82.12808	I	This study
OQ656597	SRQP11	Sydenham	Great Lakes	Ontario	42.5892	-82.12808	I	This study
OQ656598	SRQP12	Sydenham	Great Lakes	Ontario	42.5892	-82.12808	I	This study
OQ656599	SRQP13	Sydenham	Great Lakes	Ontario	42.5892	-82.12808	I	This study
OQ656600	SRQP14	Sydenham	Great Lakes	Ontario	42.5892	-82.12808	I	This study
OQ656601	SRQP17	Sydenham	Great Lakes	Ontario	42.5892	-82.12808	I	This study
OQ656602	SRQP18	Sydenham	Great Lakes	Ontario	42.5892	-82.12808	I	This study
OQ656603	SRQP19	Sydenham	Great Lakes	Ontario	42.5892	-82.12808	I	This study
OQ656604	SRQP20	Sydenham	Great Lakes	Ontario	42.5892	-82.12808	I	This study
OQ656605	TRQP01	Thames	Great Lakes	Ontario	42.64085	-81.7031	I	This study
OQ656606	TRQP02	Thames	Great Lakes	Ontario	42.64085	-81.7031	I	This study
OQ656607	TRQP03	Thames	Great Lakes	Ontario	42.64085	-81.7031	I	This study
OQ656608	TRQP04	Thames	Great Lakes	Ontario	42.64085	-81.7031	I	This study
OQ656609	TRQP05	Thames	Great Lakes	Ontario	42.64085	-81.7031	I	This study
OQ656610	TRQP06	Thames	Great Lakes	Ontario	42.64085	-81.7031	I	This study
OQ656611	TRQP08	Thames	Great Lakes	Ontario	42.64085	-81.7031	I	This study
OQ656612	TRQP13	Thames	Great Lakes	Ontario	42.64085	-81.7031	I	This study
OQ656613	TRQP14	Thames	Great Lakes	Ontario	42.64085	-81.7031	I	This study
OQ656614	TRQP15	Thames	Great Lakes	Ontario	42.64085	-81.7031	I	This study
OQ656615	ZAM004	Colorado River	Colorado	Texas	29.555491	-96.401673	IV	This study
OQ656616	ZAM008	Colorado River	Colorado	Texas	29.555491	-96.401673	IV	This study
OQ656617	ZAM009	Colorado River	Colorado	Texas	29.555491	-96.401673	IV	This study
OQ656618	ZAM012	Colorado River	Colorado	Texas	29.555491	-96.401673	IV	This study
OQ656619	ZAM016	Colorado River	Colorado	Texas	29.555491	-96.401673	IV	This study
OQ656620	ZAM022	Colorado River	Colorado	Texas	29.555491	-96.401673	IV	This study
OQ656621	ZAM024	Colorado River	Colorado	Texas	29.555491	-96.401673	IV	This study
OQ656622	ZAM026	Colorado River	Colorado	Texas	29.555491	-96.401673	IV	This study
OQ656623	ZAM029	Colorado River	Colorado	Texas	29.555491	-96.401673	IV	This study
OQ656624	ZAM030	Colorado River	Colorado	Texas	29.555491	-96.401673	IV	This study
OQ656625	ZAM040	Colorado River	Colorado	Texas	29.555491	-96.401673	IV	This study
